# Geriatric urinary tract infections: The value of laboratory parameters in estimating the need for bacteremia and Intensive Care Unit

**DOI:** 10.12669/pjms.35.1.97

**Published:** 2019

**Authors:** Ayse Sagmak Tartar, Safak Ozer Balin

**Affiliations:** 1 Department of Infectious Diseases and Clinical Microbiology, Faculty of Medicine, Firat University, Elazig, Turkey; 2 Department of Infectious Diseases and Clinical Microbiology, Faculty of Medicine, Firat University, Elazig, Turkey

**Keywords:** Urinary tract infection, Intensive care, Biochemical parameters, Geriatrics

## Abstract

**Objective:**

We investigated the geriatric patients diagnosed as urinary tract infection and evaluated the effects of white blood cell (WBC), neutrophil count, platelet, mean platelet volume (MPV), red cell distribution width (RDW), total bilirubin, direct bilirubin, creatine, albumin, erythrocyte sedimentation rate, C-reactive protein, and neutrophil/lymphocyte ratio parameters on estimating the need for bacteremia and intensive care (IC) for the patients with pyelonephritis

**Methods:**

Between 2016-2017, a total number of 188 patients aged 65 years and above were retrospectively evaluated at the infectious diseases clinic.

**Results:**

The 124 (66%) of the patients were male and 64 (34%) were female. The laboratory values of the patients with pyelonephritis and urosepsis were found to be significantly lower in only RDW bacteremic patients (p=0.047). The laboratory values during the application of third-step IC unit patients, who were treated and discharged, were compared. Albumin was significantly lower, while direct bilirubin, AST and ALT were significantly higher (p<0.05).

**Conclusions:**

Patients, whose biochemical parameters have changed, especially during admission or follow-up, should be evaluated carefully in terms of urosepsis, multiple organ failure and IC need. A number of diagnostic tests have been described to predict the need for sepsis and IC. However, many of them cannot be performed in emergency conditions. It is a great advantage that the parameters we use in our work are easily accessible and can be performed in emergency conditions.

## INTRODUCTION

One of the most common infection among elderly people is the urinary tract infection (UTI). The prevalence of the complicated UTI including bacteremia increases with age.^1^,^2^ The prevalence is high among elders with diabetes. It predisposes some serious complications with other diseases such as xanthogranulomatous pyelonephritis, perinephric abscess, emphysematous, renal abscess and UTI.^3^,^4^ Delayed diagnosis can be seen due to the atypical symptoms.^5^

Determining complete blood counts is practical and cost-effective, and it includes parameters important for several diseases. For example, red cell distribution width (RDW) shows the heterogeneity of circulating erythrocytes. Large cohort studies have demonstrated a positive correlation of RDW levels with inflammation^6^ and infectious diseases.^7^

Mean platelet volume (MPV) is presented in the complete blood cell count, which is routinely used in emergency departments. The size of the platelet is correlated with the activity and the function of the platelet; larger platelets are more active than small ones. Thus, MPV may be used as a biomarker in inflammatory disorders, sepsis-like conditions.^8^

In this study, we investigated the geriatric patients admitted to our clinic with the diagnosis of UTI and evaluated the effects of white blood cell (WBC), neutrophil count, platelet, MPV, RDW, total bilirubin, direct bilirubin, creatine, albumin, erythrocyte sedimentation rate (ESR), C-reactive protein (CRP), and neutrophil/ lymphocyte ratio parameters on estimating the need for bacteremia and intensive care (IC) for the patients with pyelonephritis.

## METHODS

This retrospective study has been conducted in accordance with the principles of the Helsinki Declaration and approved by the local Institutional Review Board (5.1.2018 / Decision number 1/3).

Between June 2016 and December 2017, patients aged 65 years and above were retrospectively evaluated at the infectious diseases clinic. In the first stage, the patients who were diagnosed with UTI were included in the study. Demographic data, concomitant co-morbid conditions, urine and blood culture results were evaluated. In the second stage, 19 patients with double infection focus were excluded from the study. Laboratory parameters at the admission day were evaluated according to the diagnosis.

In the third stage, the values of WBC, neutrophil count, platelet, MPV, RDW, total bilirubin, direct bilirubin, creatine, albumin, erythrocyte sedimentation rate, CRP, and neutrophil/ lymphocyte ratio parameters were compared in order to predict the risk of bacteremia and third stage intensive care unit (ICU) among patients with pyelonephritis and urosepsis. Due to the acceptance of cystitis as a local infection, and the low number of patients with pyonephrosis and epididiymo-orchitis, those patients were also excluded from the study in the third stage.

### Statistical Analysis

Data were analyzed using the IBM Statistical Package for Social Sciences v22 (SPSS, Inc., Chicago, IL, USA). Descriptive statistics, such as frequencies or percentages for categorical variables and mean (±standard deviation) and median+ interquartile range (IQR) for continuous variables, were used to describe baseline demographic data and clinical characteristics. The variables were investigated using visual (histograms) and analytic methods (Shapiro-Wilk’s test) to determine whether or not they are normally distributed. The Mann-Whitney U test or Student’s t-test were applied to compare continuous variables, depending on the normality of the data distribution. The *p*-values <0.05 were considered to be statistically significant for all analysis.

## RESULTS

Between 2016-2017, a total number of 188 patients over 65 years of age were admitted to our clinic because of UTI. The 124 (66%) of the patients were male and 64 (34%) were female. The median age was 78 (interquartile range: 75-84). Among patients diagnosed with UTIs, 119 (63.3%) had pyelonephritis, 54 (28.7%) had cystitis, 7 (3.7%) urosepsis, 5 (2.7%) had prostatitis, 2 (1.1%) had epididymo-orchitis, one had pyonephrosis. Another infectious focus was found in 19 patients. There were pneumonia in 14 (7.4%) patients, soft tissue infection in 3 (1.6%), mucormycosis in 1 (0.5%), and spondylodiscitis in 1 (0.5%) together with UTI.

No additional comorbid status was found in 18 (9.6%) patients. There were hypertension in 73 (42.9%) patients, diabetes mellitus in 65 (38.2%), benign prostatic hyperplasia in 48 (28.2%), malignite in 43 (25.2%), ischemic cerebrovascular disease in 30 (17.6%), chronic renal failure in 22 (12.9%), alzheimer in 21 (12.3%), and nephrolithiasis in 10 (5.8%) patients.

When the results of urine culture were examined, 17 (9%) patients had no reproduction. *Escherichia coli* *(E.coli*) reproduced in 117 (68.4%) patients, *Klebsiella spp. in* 33 (19.3%), *Pseudomonas aeruginosa* (*P. aeruginosa*) in 8 (4.7%), *Candida spp*. in 5 (2.9%), *Enterococcus spp*. in 3 (1.8%). *Proteus mirabilis* in 2 (1.2%), *Staphylococcus aureus* in 2 (1.2%), and *Coagulase- negative Staphylococci* in 1 (0.6%) patient.

The blood culture of 21 (11.1%) patients produced the same microorganism as the urine culture. *E.coli* reproduced in the blood culture of 16 (76.2%) patient, *Klebsiella spp*. in 3 (14.3%), *P. aeruginosa* in 1 (4.8%), and *Enterococcus spp. in* 1 (4.8%). The 174 (92.6%) patients were discharged with healing and 14 (7.4%) were transferred to ICU.

After the 19 patients with double infection focus were excluded, the various laboratory parameters determined on the application day were presented in Table-1.

**Table-I:** Various laboratory parameters determined on the admission day of patients (Median+ IQR).

Diagnosis	Pyelonephritis	Urosepsis	Cystitis	Prostatitis
WBC (mm^3^)	10850 (7655-14550)	9300 (8690-23630)	7100 (5850- 8590)	7740 (6500-11335)
Hb (g/dL)	11.9 (10.4-13.2)	12.6 (10.4-14.6)	12.7 (11.4-13.9)	12.1 (9.9-13.2)
Platelet (mm^3^)	248 (197-340)	191 (114-261)	248 (197-342)	221 (188-310)
AST (U/L)	25 (18-39)	25 (18-39)	25 (18-39)	20 (18-25)
ALT (U/L)	18 (13-26)	20 (14-265)	15 (11-21)	17 (13-25)
Urea	58 (42-82)	72 (53-129)	48 (37-58)	53 (31-64)
Creatinine	1.2 (0.9-1.6)	2 (0.7-2.5)	0.9 (0.8-1.4)	1.4 (1-1.7)
ESR (mm/h)	51 (31- 69)	35 (24-81)	32 (22-45)	56 (47-82)
CRP (mg/L)	92 (37-132)	73 (25-209)	11 (4.7-27)	19 (14-34)
Albumin (g/dl)	3.5 (3.2-3.9)	3.2 (2.9-3.3)	4 (3.8-4.3)	3.8 (3.5-4.1)

Hb; Hemoglobin, AST; Aspartate aminotransferase, ALT; Alanine aminotransferase

The laboratory values of patients with pyelonephritis and urosepsis with and without bacteremia were compared and found to be significantly lower in only RDW bacteremic patients (p=0.047) (Table-2).

**Table-II:** Comparison of laboratory values during the application of patients with pyelonephritis and urosepsis who had and did not have bacteremia (Median+IQR).

	Patients with bacteremia	Non-bacteremic patients	p values
WBC (mm^3^)	17300 (9450-278509)	12000 (9875-21855)	0.657
Neutrophil	13300 (7295-22700)	11000 (8165- 18500)	0.858
Plt (mm^3^)	279 (185-349)	353 (184- 536)	0.102
MPV	8.5 (8.1-9)	8.4 (8.1-9.2)	0.882
RDW	13.4 (13.1-15.1)	15.8 (14.3-17.3)	0.047*
Neutrophil/ lymphocyte ratio	12.5 (3.8-31)	14.1 (5.1-30.6)	0.139
CRP (mg/L)	159 (46-221)	104 (54-181)	0.131
ESR (mm/h)	54 (43-93)	48 (36- 81)	0.288
Albumin (g/dl)	3.3 (3-3.8)	2.8 (2.4- 3.6)	0.273
Total bilirubin	0.6 (0.5-0.8)	0.9 (0.5-0.1)	0.260
Direct bilirubin	0.2 (0.1-0.4)	0.3 (0.2-0.5)	0.470
Creatinine	1.5 (0.9-2.4)	1.3 (0.5-1.6)	0.074
AST (U/L)	28 (17-60)	41 (22-71)	0.307
ALT (U/L)	20 (18-23)	19 (13-47)	0.187

* ; p<0.05.

The 20 (95.2%) bacteremic patients were discharged after healing, and one (4.8%) patient were transferred to ICU. There was no statistically significant differences between bacteremia and being sent to ICU (p=0.684). The 4 (57.1%) of urosepsis patients were discharged with healing, and 3 (42.9%) were transferred to ICU. Being sent to ICU was significantly higher according to the patients with pyelonephritis (p=0.01). In comparison to the laboratory values of patients with urosepsis and pyelonephritis requiring third-step IC and patients with urosepsis and pyelonephritis discharged with healing; in patients who require intensive care, while albumin was significantly lower, direct bilirubin, AST and ALT were significantly higher (Table-3). There was no significant differences between the two groups in terms of other parameters.

**Table-III:** The laboratory values during the application of patients who required third-stage IC and who were discharged with healing (Median+ IQR).

	Patients with pyelonephritis and urosepsies transferred to the ICU-level 3	Pyelonephritis and urosepsis patients discharged with healing	p values
WBC (mm^3^)	10430 (6850-14837)	10620 (7700-14600)	0.789
Neutrophil	8465 (5427-12000)	8200 (5410-11000)	0.809
Plt (mm^3^)	231 (185-313)	247 (191-347)	0.711
MPV	8.9 (7.8-8.4)	8.6 (7.9-9.2)	0.791
RDW	15.2 (13.3-17)	15.6 (14.2-16.7)	0.589
Neutrophil/ lymphocyte ratio	7.5 (4.2-25.7)	6.07 (3.7-11.8)	0.400
CRP (mg/L)	73 (55-131)	92 (35-132)	0.773
ESR (mm/h)	31 (20.3-57)	52 (31-71)	0.103
Albumin (g/dl)	3.2 (2.5-3.3)	3.6 (3.2-4)	0.004*
Total bilirubin	0.9 (0.4-1.5)	0.6 (0.4-0.9)	0.151
Direct bilirubin	0.5 (0.7-0.2)	0.2 (0.1-0.3)	0.005*
Creatinine	1.3 (0.5-1.8)	1.2 (0.9-1.7)	0.753
AST (U/L)	46 (27-129)	24 (18-37)	0.004*
ALT (U/L)	28 (16-109)	18 (12-23)	0.008*

* ; p<0.05.

## DISCUSSION

The geriatric population suffers a lot because of complicated UTI. Performing indwelling catheters and disorders which avoid the bladder to completely get empty, is the major complicating factor. The UTI is seen more frequently among elders in both sex. In our study, the proportion of male patients was higher than female patients. The complication of injections of the urinary system in older men is an indication for hospitalization more frequently.

Diabetes is diagnosed in the 30% of the patients with UTI in the hospital.^9^ In a study of diabetic and non-diabetic subjects within the 55-75 years old, the incidence of UTI was 12.2 for 100 patient-years in diabetics and 6.7 in non-diabetics.^10^-^11^ A strong association between UTI, urinary instrumentation and DM was reported by Mahesh et al.^12^ The susceptibility for infections and the hospitalisation risk is increased with invasive procedures, multiple medical comorbidities, age related immunity changes, short and long-term urinary catheterisations.^12^ No additional comorbid status was detected in the 18 (9.6%) patients.

As with any ages, *E.coli* is the most frequent factor in elderly individuals. In a multi-center study of 611 cases from Turkey, the most frequently isolated agent for community-acquired complications and non-complicated complications was found as *E.coli* (71% and 90%, respectively).^13^ Also, in a hospital-related study reported from Turkey, where the mean age was 60, *E.coli* (40.8%), *Candida spp*. (23%), *Enterococcus spp*. (11%), *Pseudomonas aeruginosa* (7.6%) and *Klebsiella pneumonia* (6.8%) were found actively.^14^ In our study, *E. coli* and *Klebsiella pneumoniae w*ere identified as active agents, which is in accordance with the literature.

In case the presence of pyelonephritis with obstructive uropathy, renal abscess, perinephric abscess, emphysematous pyelonephritis, or emphysematous cystitis, bacteremia is seen very frequently and the infection may be fatal. The prevalence of complicated UTI including bacteremia increases with age. In a series of an older study, bacteremia was seen with a rate of 61% for elderly patients who were hospitalized with pyelonephritis. The incidence of shock was high.^15^ In this study, bacteremia was present in 21 patients.

Lower tract infection of elder patients with complicated UTI can generally be managed as the setting of outpatients. However, unless the patient is minimally ill, upper tract infection of elder individuals can be managed as inpatients. Only RDW bacteria were found to be significantly lower in patients with pyelonephritis and urosepsis compared to the laboratory values during the application (p=0.047). Extensive cohort studies report a positive correlation in RDW levels with inflammation^6^ and infectious diseases such as acute pancreatitis, sepsis and septic shock.^7^ This may be due to the fact that our patient population is in the geriatric age group. RDW increases especially in patients with iron deficiency anemia. The prevalence of iron deficiency anemia in elder patients may limit the use of RDW.

Urinary tract infections in geriatric patients may be mild, or may result in bacteremia, sepsis or death.^16^ Meyers et al. evaluated 100 bacterial episodes over 65 years of age in their study and they found that the genitourinary was the most common source of bacterium (27%).^17^ In another study where bacteremic UTIs were evaluated, mortality rate was 16.1%.^16^ In our study, no statistically significant correlation was found between the bacteremia and the transfer to the ICU (p=0.684). In patients with urosepsis, the rate of ICU transfer was significantly higher than in patients with pyelonephritis (p=0.01). In our study, albumin was significantly lower in patients requiring IC, whereas direct bilirubin, AST and ALT were significantly higher. In older patients, the diagnosis may be delayed due to the faint symptoms and the disease may progress to the urosepsis. In this respect, clinician should be more careful for geriatric patients and should evaluate urosepsis.

### Limitations of the study

It is its retrospective design and being a single-site study. Prospective and multi-site studies could provide more certain results.

## CONCLUSION

As a result, UTIs in geriatric patients may be confronted with different situations and their rate of bacteremia is high. Patients whose biochemical parameters have changed, especially during admission or follow-up, should be evaluated carefully in terms of urosepsis, multiple organ failure and IC need. A number of diagnostic tests have been described to predict the need for sepsis and IC. However, many of them cannot be performed in emergency conditions. It is a great advantage that the parameters we use in our work are easily accessible and can be performed in emergency conditions.

### Author’s Contribution

**AST and SOB:** Designed and performed the study.

**AST:** Did data collection and writing of manuscript.

**AST:** Did statistical analysis and editing of manuscript.
